# Artificial Intelligence for the Detection of Small Bowel Lesions and Neoplasia: A Scoping Review

**DOI:** 10.7759/cureus.112283

**Published:** 2026-07-08

**Authors:** Faure Rodriguez Velasquez, Andres Montoya, Daniela Riaño-Pineda, Gabriela Urdinola, Alejandra Sogamoso-Bohórquez, Juan Galves-Cetina, Eduardo Tuta-Quintero

**Affiliations:** 1 Gastroenterology, Universidad de La Sabana, Bogotá, COL; 2 Gastroenterology, EndoGut Research Group, Bogotá, COL; 3 Gastroenterology, Fundación Clínica Shaio, Bogotá, COL; 4 Internal Medicine, Universidad de La Sabana, Bogotá, COL; 5 Medicina Interna-Oncología, Hospital Militar Central, Bogotá, COL; 6 Epidemiology and Public Health, Universidad de La Sabana, Bogotá, COL

**Keywords:** artificial intelligence, cancer, endoscopy, small bowel, systematic review

## Abstract

Artificial intelligence (AI) has emerged as a promising tool for improving the detection and characterization of small bowel (SB) lesions through endoscopic imaging. This scoping review mapped and synthesized the available evidence on the diagnostic performance of AI systems applied to capsule endoscopy (CE) and device-assisted enteroscopy for identifying SB lesions with potential malignant or premalignant relevance. The review was conducted according to the methodological frameworks of Arksey and O’Malley, Levac, and the Joanna Briggs Institute, and was reported in accordance with Preferred Reporting Items for Systematic reviews and Meta-Analyses extension for Scoping Reviews (PRISMA-ScR) recommendations. Searches were performed in PubMed, Scopus, and Embase, including original studies that evaluated AI systems on SB endoscopic images or videos and reported quantitative diagnostic metrics.

A total of 13 studies were included, most of them retrospective in design. The majority originated from China and Japan, and capsule endoscopy was the most commonly used imaging modality. Automated lesion detection represented the principal application of AI, often combined with diagnostic classification tasks. Most models were based on convolutional neural networks, including architectures such as ResNet50, Single Shot Multibox Detector, YOLOv5, and nnU-Net. Across studies, diagnostic performance was consistently high, with sensitivities ranging from 81.2% to 98.6%, specificities from 88.6% to 99.8%, and area under the ROC curve values close to 1.0 in several reports. AI appears to improve the detection of SB lesions and significantly reduce endoscopic reading times, particularly in capsule endoscopy. However, the current evidence remains insufficient to support the use of AI as a screening tool for SB cancer, as most available studies are retrospective and lack robust prospective multicenter validation.

## Introduction and background

The small bowel (SB) accounts for approximately 75% of the total length of the gastrointestinal tract [1,2]. However, neoplasms arising in this segment are uncommon, representing only about 3% of all gastrointestinal cancers [3,4]. Despite their low incidence, SB neoplasms pose a significant diagnostic challenge due to their nonspecific clinical presentation, the complex anatomy of the SB, and the lack of established screening strategies, all of which frequently result in delayed diagnosis at advanced stages [1,3-5]. Adenocarcinoma is the most common histological subtype of SB cancer, accounting for approximately 40% of cases, followed by neuroendocrine tumors and lymphoproliferative neoplasms [4,5].

The diagnostic evaluation of the SB requires a multimodal approach. Upper gastrointestinal endoscopy is useful for detecting duodenal lesions, whereas colonoscopy allows evaluation of the terminal ileum [5,6]. Capsule endoscopy (CE) plays a central role in the assessment of jejunal and ileal pathology by enabling visualization of nearly the entire SB mucosa [6-8]. Cross-sectional imaging techniques, including computed tomography enterography and magnetic resonance enterography, provide valuable information for disease staging and assessment of disease extent, whereas device-assisted enteroscopy enables tissue sampling for histopathological confirmation [7-9]. Nevertheless, these diagnostic modalities remain limited by operator dependence, the complex anatomy of the SB, the large volume of images generated, and prolonged interpretation times, all of which increase the risk of missed lesions and contribute to diagnostic variability [4,5,8-11].

In this context, artificial intelligence (AI) has emerged as a promising tool for optimizing endoscopic image interpretation by automating the analysis of large imaging datasets and recognizing complex patterns, with the potential to improve diagnostic performance while reducing reading time [12-15]. Current AI applications in SB endoscopy have focused on the automated detection, classification, localization, and characterization of a wide spectrum of lesions, including vascular, inflammatory, and protruding abnormalities [10-15]. However, although some of these findings may represent early manifestations of neoplastic processes, it remains unclear to what extent existing AI systems have been specifically developed and validated for the detection and characterization of malignant and premalignant SB lesions [12-15]. Furthermore, the available evidence remains fragmented across different imaging modalities, deep learning algorithms, and diagnostic objectives, with most studies being retrospective and limited by a lack of prospective and external validation, making it difficult to define the clinical role of these technologies [10-15]. Therefore, this scoping review aimed to analyze and synthesize the available literature on the use of AI in SB endoscopy for detecting and characterizing malignant and premalignant lesions.

Methods

Scoping Review Design

This scoping review was conducted according to the methodological framework proposed by Arksey and O’Malley [16], further refined by Levac et al. [17], and guided by the Joanna Briggs Institute recommendations for scoping reviews [18]. Reporting followed the Preferred Reporting Items for Systematic Reviews and Meta-Analyses extension for Scoping Reviews (PRISMA-ScR) [19]. The review protocol, entitled Artificial intelligence for the detection of small bowel lesions and its potential implications for neoplasia detection: a scoping review, was prospectively registered with the Open Science Framework (OSF) on June 27, 2026. 

Formulation of the Research Question

The research question was developed using the Population, Concept, and Context (PCC) framework. The population included patients undergoing SB endoscopic evaluation for suspected or confirmed lesions with malignant or premalignant relevance, including SB tumors, polyps, protruding lesions, ulcerative lesions, adenocarcinoma, neuroendocrine tumors, lymphoma, and other abnormalities relevant to the differential diagnosis or early recognition of neoplastic disease. The concept included artificial intelligence systems based on machine learning, deep learning, convolutional neural networks, object detection models, segmentation models, or computer-aided diagnosis. The context was limited to endoscopic images or videos obtained via CE and device-assisted enteroscopy, as these are the main modalities used for direct SB mucosal evaluation. The guiding research question was: What is the diagnostic performance of AI systems applied to CE and device-assisted enteroscopy images or videos for the detection, characterization, classification, or segmentation of premalignant and malignant SB lesions?

Search Strategy

Searches were performed in PubMed, Scopus, and Embase. The search strategies were revised and harmonized to improve alignment with the review objective and to focus specifically on AI applications in small bowel endoscopy. The updated strategy combined terms related to AI, machine learning, deep learning, convolutional neural networks, computer-aided detection, CE, video CE, SB capsule, device-assisted enteroscopy, double-balloon enteroscopy, single-balloon enteroscopy, spiral enteroscopy, jejunum, ileum, SB tumors, adenocarcinoma, neuroendocrine tumors, lymphoma, polyps, protruding lesions, ulcerative lesions, premalignant lesions, and malignant lesions. Broader lesion-related terms were retained only when clinically relevant to SB neoplasia or when they reflected categories commonly used in AI-based SB lesion detection studies. The complete search strategies for each database are presented in Table 1.

**Table 1:** Search strategies (search updated February 1, 2026)

PubMed/MEDLINE
("Artificial Intelligence"[Mesh] OR "artificial intelligence"[tiab] OR AI[tiab] OR "machine learning"[tiab] OR "deep learning"[tiab] OR "deep neural network*"[tiab] OR "neural network*"[tiab] OR "convolutional neural network*"[tiab] OR CNN[tiab] OR "computer-aided detection"[tiab] OR "computer aided detection"[tiab] OR "computer-aided diagnosis"[tiab] OR "computer aided diagnosis"[tiab] OR CADe[tiab] OR CADx[tiab] OR "clinical decision support"[tiab]) AND ("Endoscopy, Gastrointestinal"[Mesh] OR endoscop*[tiab] OR gastroscop*[tiab] OR esophagogastroduodenoscop*[tiab] OR EGD[tiab] OR "Capsule Endoscopy"[Mesh] OR "capsule endoscop*"[tiab] OR "video capsule"[tiab] OR VCE[tiab] OR SBCE[tiab] OR enteroscop*[tiab] OR "device-assisted enteroscopy"[tiab] OR "balloon enteroscopy"[tiab] OR "double-balloon enteroscopy"[tiab] OR DBE[tiab] OR "single-balloon enteroscopy"[tiab] OR SBE[tiab] OR "spiral enteroscopy"[tiab]) AND ("small bowel"[tiab] OR "small intestine"[tiab] OR enter*[tiab] OR jejun*[tiab] OR ileum[tiab] OR ileal[tiab] OR duoden*[tiab]) Filters: from 1000/1/1 - 2026/6/27 Sort by: Publication Date
Scopus
TITLE-ABS-KEY ( "artificial intelligence" OR "machine learning" OR "deep learning" OR "neural network*" OR "convolutional neural network*" OR "computer aided detection" OR "computer aided diagnosis" ) AND TITLE-ABS-KEY ( "small bowel" OR "small intestine" OR enter* OR jejun* OR ileum OR ileal OR duoden* ) AND TITLE-ABS-KEY ( endoscop* OR enteroscop* OR "capsule endoscopy" OR "capsule endoscop*" OR "video capsule" OR "device assisted enteroscopy" OR "balloon enteroscopy" OR "double balloon enteroscopy" OR "single balloon enteroscopy" OR "spiral enteroscopy" ) AND TITLE-ABS-KEY ( neoplas* OR cancer* OR tumor* OR tumour* OR adenocarcinoma* OR adenoma* OR polyp* OR dysplasia OR premalign* OR precancer* OR lymphoma* OR "neuroendocrine tumor*" OR "gastrointestinal stromal tumor*" OR GIST ) AND PUBYEAR < 2026
Embase
('artificial intelligence'/exp OR 'artificial intelligence':ti,ab,kw OR 'machine learning'/exp OR 'machine learning':ti,ab,kw OR 'deep learning':ti,ab,kw OR 'neural network*':ti,ab,kw OR 'convolutional neural network*':ti,ab,kw OR 'computer aided detection':ti,ab,kw OR 'computer aided diagnosis':ti,ab,kw OR 'clinical decision support':ti,ab,kw) AND ('small intestine'/exp OR 'small bowel':ti,ab,kw OR 'small intestine':ti,ab,kw OR enter*:ti,ab,kw OR jejun*:ti,ab,kw OR ileum:ti,ab,kw OR ileal:ti,ab,kw OR duoden*:ti,ab,kw) AND ('capsule endoscopy'/exp OR 'enteroscopy'/exp OR endoscop*:ti,ab,kw OR enteroscop*:ti,ab,kw OR 'capsule endoscop*':ti,ab,kw OR 'video capsule':ti,ab,kw OR 'device assisted enteroscopy':ti,ab,kw OR 'double balloon enteroscopy':ti,ab,kw OR 'single balloon enteroscopy':ti,ab,kw OR 'balloon enteroscopy':ti,ab,kw OR 'spiral enteroscopy':ti,ab,kw) AND ('intestinal tumor'/exp OR neoplas*:ti,ab,kw OR cancer*:ti,ab,kw OR tumor*:ti,ab,kw OR tumour*:ti,ab,kw OR adenocarcinoma*:ti,ab,kw OR adenoma*:ti,ab,kw OR polyp*:ti,ab,kw OR dysplasia:ti,ab,kw OR premalign*:ti,ab,kw OR precancer*:ti,ab,kw OR lymphoma*:ti,ab,kw OR 'neuroendocrine tumor*':ti,ab,kw OR 'gastrointestinal stromal tumor*':ti,ab,kw OR GIST:ti,ab,kw) AND (1000-2026)/py

Eligibility Criteria

Original studies were included if they evaluated AI systems applied to SB endoscopic images or videos and reported quantitative diagnostic performance metrics, such as sensitivity, specificity, accuracy, area under the receiver operating characteristic curve, positive predictive value, negative predictive value, F1-score, Dice coefficient, or detection rate. Eligible endoscopic modalities included CE, SB capsule, device-assisted enteroscopy, double-balloon enteroscopy, single-balloon enteroscopy, and spiral enteroscopy.

The primary focus was malignant or premalignant SB disease, including SB tumors, adenocarcinoma, neuroendocrine tumors, lymphoma, polyps, protruding lesions with possible neoplastic relevance, and precursor or suspicious lesions. Non-neoplastic lesions, including inflammatory, ulcerative, vascular, and bleeding lesions, were included only when they were relevant to the diagnostic pathway of SB neoplasia, could mimic or overlap with neoplastic lesions, or represented lesion categories used by existing AI models that may inform early recognition of clinically significant disease. Narrative reviews, editorials, letters to the editor, conference abstracts without sufficient data, studies without quantitative diagnostic metrics, studies not involving real clinical endoscopic images or videos, and studies focused exclusively on technical algorithm development without clinical image validation were excluded.

Study Selection

All retrieved records were imported into Rayyan [20]. Duplicate records were identified and removed before screening. Two reviewers independently screened titles and abstracts according to the predefined eligibility criteria. Full-text articles were then assessed for final inclusion. Disagreements during title/abstract screening or full-text assessment were resolved through discussion and consensus, with involvement of a third reviewer when necessary. The study selection process was summarized using a PRISMA flow diagram.

Data Extraction

Data extraction was performed using a standardized extraction matrix [21]. Extracted variables included author, year of publication, country of origin, study design, target lesion or pathology, imaging modality, AI task, model architecture, dataset size, unit of analysis, validation approach, comparator, diagnostic performance metrics, main findings, and reported methodological limitations. Extracted data were checked by two reviewers to ensure consistency and accuracy. Disagreements or uncertainties during data extraction were resolved by consensus, with consultation of a third reviewer when required [21].

Definition and Classification of Artificial Intelligence Tasks

AI tasks were categorized by the primary algorithmic output and reported clinical purpose. Detection was defined as automated identification or localization of suspicious lesions in images or videos. Classification was defined as assignment of a diagnostic or lesion category after lesion recognition. Segmentation was defined as delineation of lesion boundaries or affected areas. Monitoring was defined as automated assessment of disease burden, lesion progression, or interval change over time. AI-assisted reading was defined as the use of AI to reduce reading time, prioritize frames, or support diagnostic agreement with expert readers. Studies were classified by their primary AI application. When a study included more than one task, all relevant tasks were recorded, but the primary task was determined by the study's main objective and reported outcome.

Evidence Synthesis and Analysis

A descriptive narrative synthesis was performed because of heterogeneity in study designs, imaging modalities, lesion types, AI architectures, validation approaches, units of analysis, and reported outcomes [21]. Studies were classified as focusing on malignant or premalignant lesions (e.g., neoplastic lesions, small bowel tumors, follicular lymphoma, duodenal polyps associated with familial adenomatous polyposis, and protruding lesions with suspected neoplastic potential) or non-neoplastic lesions, including inflammatory, ulcerative, vascular, or other nonspecific small bowel abnormalities. Imaging modalities were grouped as CE, device-assisted enteroscopy, or other relevant SB endoscopic modalities. AI model characteristics were grouped as convolutional neural network-based models, object detection models, segmentation models, traditional computer-aided diagnosis approaches, or other machine learning/deep learning methods. 

Evidence directly evaluating malignant or premalignant lesions was analyzed separately from evidence addressing general SB lesion detection. Quantitative synthesis was considered inappropriate due to differences in populations, lesion definitions, imaging modalities, AI tasks, model architectures, reference standards, units of analysis, validation methods, and reported performance metrics. A formal risk-of-bias or methodological quality assessment using QUADAS-2, PROBAST-AI, or similar tools was not performed [22,23]. 

## Review

Results

**Figure 1:**
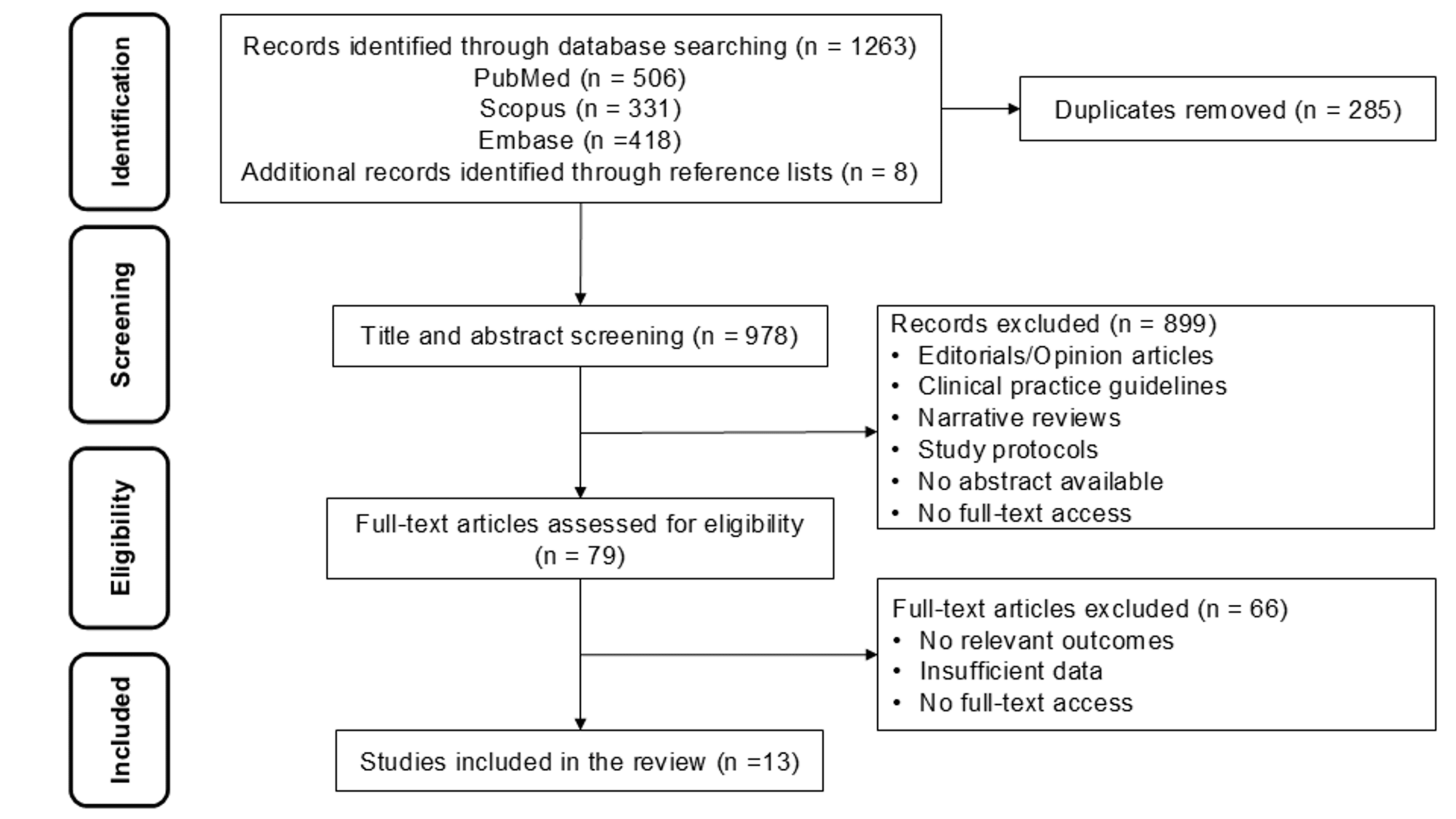
PRISMA flowchart for study selection PRISMA, Preferred Reporting Items for Systematic Reviews and Meta-Analyses.

A total of 13 studies were included (Figure 1) [24-35], with a predominance of retrospective designs (9/13; 69.2%) [24,25,27-30,33-35], followed by one prospective study (1/13; 7.7%) [32] and one pilot study (1/13; 7.7%) [31]. Most studies originated from China (4/13; 30.8%, including one international collaboration with Denmark) [24,32,35] and Japan (4/13; 30.8%) [28-30,33], followed by Portugal (3/13; 23.1%) [25-27], while Singapore [31] and the United States [34] each contributed one study (1/13; 7.7%) (Table 2). Overall, 46.2% (6/13) specifically evaluated malignant or premalignant lesions of the SB or duodenum, including neoplastic lesions [29], follicular lymphoma [33], small bowel tumors [35], duodenal polyps associated with familial adenomatous polyposis [34], and protruding lesions considered to have neoplastic potential [26,27] (Table 3). In contrast, the remaining 53.8% of studies (7/13) evaluated inflammatory, ulcerative, vascular, mixed, or nonspecific SB abnormalities, or developed AI systems for general lesion detection rather than cancer-specific applications [24,25,28,30-32]. CE was the predominant imaging modality (9/13; 69.2%) [24,27-33,35], followed by device-assisted enteroscopy (2/13; 15.4%) [25,26] and upper gastrointestinal endoscopy (1/13; 7.7%) [34].

**Table 2:** Studies evaluating artificial intelligence applications for the detection, classification, and characterization of small bowel lesions. AI, artificial intelligence; SB, small bowel; FAMCE, fully automated magnetically controlled capsule endoscopy; SBCE, small bowel capsule endoscopy; DAE, device-assisted enteroscopy; CNN, convolutional neural network; CAD, computer-aided diagnosis; SSD, Single Shot Multibox Detector; AUC, area under the curve; Sens, sensitivity; Spec, specificity; PPV, positive predictive value; NPV, negative predictive value; Acc, accuracy; FAP, familial adenomatous polyposis.

Author (Year)/Country	Study type and pathology	Imaging modality	AI task	Sample size	Main metrics	Key findings	Limitations
Xie et al. [24] 2024 China–Denmark	Retrospective study. Pathology not specified	FAMCE / SBCE	Lesion detection and recognition using deep learning (SS Plus)	1,069 examinations, 342 videos	Sensitivity: gastric 98.24%, SB 96.74%; specificity: gastric 96.49%, SB 99.21%; accuracy 97.66%	Improved sensitivity and specificity with reduced reading time	Limited number of endoscopists
Martins et al. [25] 2023 Portugal	Retrospective study. Small bowel ulcers and erosions	Device-assisted enteroscopy (DAE)	Detection and classification using CNN	6,772 images (250 exams)	Sens 88.5%, Spec 99.7%, PPV 96.4%, NPV 98.9%, Acc 98.9%, AUC 1.0	High diagnostic performance with real-time analysis capability	Single-center retrospective study
Cardoso et al. [26] 2022 Portugal	Diagnostic development and validation. Protruding lesions	DAE	Detection and classification using CNN	7,925 images (72 patients)	Sens 97.0%, Spec 97.4%, PPV 94.6%, NPV 98.6%, AUC 1.0	High sensitivity reduces missed lesions	Retrospective study with limited sample
Mascarenhas et al. [27] 2022 Portugal	Retrospective study. Protruding lesions (P1/P2)	Capsule endoscopy	Detection, characterization, and classification (CNN)	1,229 patients, 18,625 images	Sens 96.8%, Spec 96.5%, AUC up to 1.0	High ability to detect and differentiate lesions based on bleeding risk	Single-center study with static images; limited generalizability
Hosoe et al. [28] 2022 Japan	Retrospective study. Clinically significant small bowel lesions	Capsule endoscopy	Automated detection with CNN (segmentation)	172 cases	Sens 93.4%, Spec 97.8%, AUC 0.99	Reduced images for review and reading time (~10 min); higher performance for bleeding and angiodysplasia	Lower performance in polyps/lymphoma; lack of prospective validation
Aoki et al. [29] 2021 Japan	Multicenter retrospective study. Neoplastic and vascular lesions	Capsule endoscopy	Detection and classification using CNN	379 videos (5M images)	Overall detection 99% vs 89% (QuickView)	Improved diagnostic performance and reduced reading time without loss of detection	Patient-level analysis; limited generalizability
Aoki et al. [30] 2019 Japan	Development and validation. Erosions and ulcerations	Capsule endoscopy	Automated detection (CNN SSD)	10,440 images (validation)	Sens 88.2%, Spec 90.9%, AUC 0.958	High accuracy with ultra-fast processing (0.02 s/image)	Static images; no etiological differentiation
Jiang et al. [31] 2024 Singapore	Pilot study. Multiple small bowel pathologies	Capsule endoscopy	Classification and detection (CNN, ResNet50)	29 patients, 36,156 images	AUC-ROC 0.969; Top-1 acc 84%	Best performance with ResNet50; potential to reduce diagnostic time	Small sample; trained on static images
Li et al. [32] 2024 China	Development and prospective study. Small bowel lesions	Capsule endoscopy	Detection and localization (YOLOv5)	298 patients (prospective phase)	Sens 94.0%, Spec 92.3%, Acc 93.1%	Performance comparable to experts with significant reduction in reading time	Limited clinical validation
Sumioka et al. [33] 2023 Japan	Retrospective study. Follicular lymphoma	Capsule endoscopy	Detection and monitoring (CNN)	26 patients, 213,574 images	Sens 81.2%, Spec 88.6%, AUC 0.91	Useful for objective disease monitoring	Small sample; dependent on bowel preparation quality
Schupack et al. [34] 2024 USA	Retrospective study. Duodenal polyps (FAP)	Upper GI endoscopy	Detection and segmentation (nnU-Net)	870 images	Detection 78.6%, Dice 0.73	Promising prototype for polyp detection	Static images; lower performance with high polyp burden
Li et al. [35] 2011 China (Hong Kong)	Development and validation. Small bowel tumors	Capsule endoscopy	Detection and classification (CAD)	1,200 images (10 patients)	Sens 92.33%, Spec 88.67%, Acc 90.50%	Promising results using wavelet+LBP and ensemble classifiers	Small sample; static images; requires further validation

**Table 3:** Classification of included studies according to lesion type † Li et al. [32] evaluated multiple small bowel lesion types, including protruding lesions, but the AI model was developed for general lesion detection rather than specifically targeting malignant or premalignant disease.

Lesion category	No. of studies (%)	Studies	Pathologies included
Malignant	3 (23.1%)	Aoki et al. [29], Sumioka et al. [33], Li et al. [35]	Neoplastic lesions, follicular lymphoma, small bowel tumors
Premalignant	3 (23.1%)	Cardoso et al. [26], Mascarenhas et al. [27], Schupack et al. [34]	Protruding lesions with suspected neoplastic potential; duodenal polyps associated with familial adenomatous polyposis
Other small bowel lesions	7 (53.8%)	Xie et al. [24], Martins et al. [25], Hosoe et al. [28], Aoki et al. [30], Jiang et al. [31], Li et al. [32]†	Inflammatory, ulcerative, vascular, mixed, or nonspecific lesions; AI systems for general lesion detection

Automated lesion detection was the main application of AI, reported in most studies (11/13; 84.6%) [24-33,35], frequently combined with diagnostic classification tasks (8/13; 61.5%) [25-27,29,31,32,35]. Segmentation was evaluated in two studies (2/13; 15.4%) [28,34], mainly for delineating lesion boundaries or quantifying polyps. Other specific applications, such as bowel preparation assessment [31], multi-label detection [32], and automated disease monitoring [33], were reported in individual studies. Most models were based on convolutional neural networks, including architectures such as ResNet50 [31], Single Shot Multibox Detector [30], YOLOv5 [32], and nnU-Net [34]. Diagnostic performance was consistently high, with sensitivities ranging from 81.2% to 98.6%, specificities from 88.6% to 99.8%, and area under the ROC curve values approaching 1.0 in several studies [24-32,35]. Additionally, multiple studies reported significant reductions in reading time, near real-time processing, and high agreement with expert endoscopists [24,27-30,32].

Xie et al. [24] reported that Saliency Segmentation-based Pattern Recognition significantly improved performance in the interpretation of CE videos for intestinal pathology, increasing sensitivity from 84.19% to 96.74%, specificity from 98.43% to 99.21%, and diagnostic accuracy from 89.47% to 97.66%. These findings suggest that this approach may help reduce the performance gap between less experienced and expert endoscopists in CE interpretation. Martins et al. [25] demonstrated that a convolutional neural network-based algorithm achieved high performance in the automatic detection of ulcerative lesions using device-assisted enteroscopy, with a sensitivity of 88.5%, specificity of 99.7%, and overall diagnostic accuracy of 98.9%. Cardoso et al. showed that a convolutional neural network for the detection of protruding lesions using device-assisted enteroscopy achieved a sensitivity of 97.0%, specificity of 97.4%, positive predictive value of 94.6%, negative predictive value of 98.6%, and an overall accuracy of 97.3%, with an area under the curve of 1.00 [26]. Furthermore, the model processed the validation dataset in 10 seconds, equivalent to 157 images per second.

Hosoe et al. reported that their convolutional neural network-based algorithm for CE achieved a global sensitivity of 93.4% and a specificity of 97.8% in detecting clinically significant small bowel lesions, with particularly high performance for bleeding and angiodysplasia, reaching sensitivities of 100% for both [28]. Additionally, the system reduced the number of images requiring review to 7.8% in the absence of massive bleeding, suggesting a reduction in reading time to approximately 10 minutes. Aoki et al. demonstrated that a convolutional neural network for the automatic detection of small bowel erosions and ulcerations via CE achieved a sensitivity of 88.2%, specificity of 90.9%, accuracy of 90.8%, and an area under the curve of 0.958, analyzing 10,440 images in 233 seconds (approximately 0.02 seconds per image), supporting its potential for rapid and efficient reading [30].

Li L et al. developed a deep learning algorithm based on a real-time object detection model adapted for CE, capable of identifying and localizing lesions in both images and videos [32]. In video analysis, the model achieved sensitivities of 91.9% for vascular lesions, 92.2% for ulcerative or erosive lesions, 91.4% for protruding lesions, 93.1% for parasites, 93.3% for diverticula, 95.1% for active bleeding, and 100% for villous lesions. Overall, it demonstrated a sensitivity of 94.0%, specificity of 92.3%, and accuracy of 93.1%, with a significantly reduced reading time compared to both experts and non-experts (5.62 ± 2.81 minutes), suggesting its potential as a reliable tool to support automated detection of small bowel lesions in clinical practice.

Discussion

This scoping review mapped the available evidence on AI applications in SB endoscopy, with a particular focus on malignant and premalignant lesions. Although AI demonstrated consistently high diagnostic performance across the included studies, the available evidence predominantly supports the automated detection of general SB abnormalities rather than cancer-specific diagnosis or characterization. Notably, few of the included studies evaluated malignant or premalignant lesions of the SB or duodenum [26,27,29,33-35]. Consequently, the potential role of AI in the early detection of SB malignancies should be interpreted cautiously, as current evidence remains limited and largely indirect.

An important finding of this review is the discrepancy between the growing number of AI applications developed for SB endoscopy and the relatively limited evidence addressing lesions directly relevant to cancer diagnosis [24-35]. Most available algorithms were designed to identify abnormalities irrespective of their underlying pathology, including inflammatory, ulcerative, vascular, mixed, or nonspecific lesions, with comparatively few studies specifically targeting neoplastic or premalignant disease [24,25,28,30-32]. In contrast, only six studies focused on lesions with malignant or premalignant potential, including protruding lesions, neoplastic lesions, follicular lymphoma, duodenal polyps associated with familial adenomatous polyposis, and small bowel tumors [26,27,29,33-35]. Therefore, the excellent diagnostic performance reported across several studies [24-32,35] should not be interpreted as evidence that current AI systems can reliably differentiate benign from premalignant or malignant SB lesions. Cancer-specific diagnosis requires not only lesion detection but also accurate lesion characterization, risk stratification, and validation against robust clinical reference standards, including histopathological confirmation whenever feasible [36-38].

Differences in AI performance across lesion types likely reflect both the visual characteristics of the lesions and the composition of the datasets used for model development [39,40]. Algorithms generally achieved excellent performance for lesions with distinctive morphological or color features, such as active bleeding, angiodysplasia, ulcerative lesions, and some protruding lesions [24-32]. In contrast, evidence for malignant and premalignant lesions remains limited [26,27,29,33-35]. Neoplastic lesions, including small bowel tumors, follicular lymphoma, and duodenal polyps associated with familial adenomatous polyposis, are often less prevalent, exhibit greater morphological heterogeneity, and may closely resemble benign protruding or inflammatory abnormalities, making automated characterization inherently more challenging. Furthermore, the relatively small number of histologically confirmed neoplastic lesions available for model training may contribute to class imbalance and reduce model robustness to these clinically important entities [5,11,40].

Consequently, the current evidence supports the use of AI primarily as a tool for automated lesion detection rather than for cancer-specific diagnosis or histopathological prediction [24-35]. While the identification of suspicious abnormalities may facilitate earlier endoscopic evaluation, biopsy, or follow-up, this potential clinical benefit remains a hypothesis rather than an established outcome. Future AI models should therefore move beyond general abnormality detection toward accurate differentiation of benign, premalignant, and malignant lesions using large, multicenter datasets with histopathological confirmation and external validation [11,40].

CE was the predominant imaging modality across the included studies [24,27-33,35], reflecting its central role in the evaluation of small bowel pathology and the substantial image burden generated during each examination. In this setting, AI has primarily been developed to assist automated lesion detection, prioritize abnormal frames, and reduce reading time [24,27-32,35]. In contrast, evidence for device-assisted enteroscopy (DAE) remains limited [25,26]. Unlike CE, DAE enables targeted lesion assessment, tissue sampling, and therapeutic intervention, suggesting that future AI applications in this setting may extend beyond lesion detection to include real-time lesion characterization, biopsy guidance, and procedural decision support [41-43]. Because CE and DAE differ substantially in image acquisition, operator dependence, diagnostic purpose, and reference standards, their diagnostic performance should be interpreted within the context of each modality rather than compared directly.

Although several studies reported sensitivities and specificities exceeding 90% and area under the curve values approaching 1.0 [24-32,35], these results should be interpreted cautiously. Most studies were retrospective, relied on highly selected image datasets, and used internal rather than external validation, increasing the risk of selection bias, spectrum bias, overfitting, and limited generalizability. Furthermore, most algorithms were evaluated at the image level rather than the examination or patient level, and only a small proportion specifically addressed histologically confirmed malignant or premalignant lesions [26,27,29,33-35]. Consequently, the excellent diagnostic performance reported in experimental settings may not fully reflect performance in routine clinical practice.

Despite these limitations, the available evidence suggests that AI may improve the efficiency of SB endoscopy by reducing reading time, prioritizing suspicious findings, and supporting less experienced endoscopists during image interpretation [24,27,30,32]. These benefits are currently supported by direct evidence and represent the most mature clinical application of AI in SB endoscopy. By contrast, the use of AI for cancer-specific diagnosis, histopathological prediction, or reliable differentiation between benign, premalignant, and malignant lesions remains investigational and requires prospective multicenter validation using diverse populations and histologically confirmed reference standards before routine clinical implementation can be recommended [14,44].

Overall, the available evidence indicates that AI has the greatest potential to improve workflow efficiency in SB endoscopy by reducing reading time, prioritizing suspicious findings, and assisting with lesion detection during image interpretation rather than providing cancer-specific diagnosis [24,27,30,32]. These findings are consistent with recent reviews of AI in capsule endoscopy, which identify automated lesion detection and workflow optimization as the most mature clinical applications, whereas robust evidence for neoplastic characterization remains limited. However, the reported diagnostic performance should be interpreted cautiously because most studies were retrospective, single-center, and based on selected image datasets with internal validation [24-35]. These methodological characteristics increase the risk of selection bias, spectrum bias, and overfitting, potentially overestimating real-world performance. Although prospective models such as the YOLOv5-based system have shown promising results [32], additional multicenter studies with external validation, patient-level analyses, and standardized reference standards are required before widespread clinical implementation can be recommended.

Compared with AI applications in colorectal and upper gastrointestinal endoscopy, AI in SB endoscopy remains at an earlier stage of development [45-47]. In colorectal endoscopy, AI-assisted polyp detection has been validated in multiple prospective and randomized studies and is increasingly incorporated into routine clinical practice. Similarly, AI systems for upper gastrointestinal endoscopy have demonstrated robust performance for the detection and characterization of early esophageal and gastric neoplasia [46,47]. AI applications in SB endoscopy continue to focus predominantly on general lesion detection, with relatively few studies evaluating histologically confirmed malignant or premalignant lesions [26,27,29,33-35]. 

Strengths and limitations

The review was conducted according to established scoping review methodology and reported in accordance with the PRISMA-ScR guideline. Furthermore, the synthesis explicitly distinguished studies evaluating malignant or premalignant lesions from those addressing inflammatory, vascular, ulcerative, or nonspecific SB abnormalities, providing a clearer interpretation of the evidence directly relevant to the review objective. The included studies were also categorized by lesion type, imaging modality, AI task, model characteristics, validation approach, and unit of analysis, allowing key methodological gaps and future research priorities to be identified.

Several limitations should also be acknowledged. First, although the review protocol was prospectively registered, no formal risk-of-bias assessment (e.g., QUADAS-2 or PROBAST-AI) was performed, which is consistent with the objectives of a scoping review but limits assessment of the methodological quality of individual studies [22]. Second, substantial heterogeneity in study design, lesion definitions, imaging modalities, AI architectures, reference standards, validation strategies, and reported performance metrics precluded quantitative synthesis. Third, most included studies were retrospective, single-center investigations based on selected static image datasets, increasing the likelihood of selection bias, spectrum bias, overfitting, and limited external validity. Finally, most studies reported image-level rather than examination- or patient-level outcomes, confidence intervals were inconsistently reported, and only six studies specifically evaluated malignant or premalignant lesions [26,27,29,33-35]. Therefore, the findings of this review should be interpreted as an evidence map highlighting current research trends and knowledge gaps rather than as confirmation of the clinical readiness of AI for cancer-specific diagnosis in SB endoscopy.

## Conclusions

Current evidence suggests that AI may support the detection of SB abnormalities, particularly in capsule endoscopy, where automated analysis could help reduce reading time and missed findings; however, the current level of evidence remains limited and should be interpreted with caution. Most available studies evaluated general lesion detection rather than true neoplastic characterization, histopathological prediction, or cancer-specific diagnosis, and the high diagnostic performance reported in several studies may be influenced by retrospective designs, selected or preprocessed static image datasets, image-level analyses, limited external validation, and scarce prospective multicenter evaluation, which may overestimate real-world clinical performance. 
